# Development and *In Vitro* Evaluation of Buccoadhesive Tablets of Metoprolol Tartrate

**DOI:** 10.4103/0250-474X.40349

**Published:** 2008

**Authors:** P. D. Nakhat, A. A. Kondawar, L. G. Rathi, P. G. Yeole

**Affiliations:** Department of Pharmaceutics, Institute of Pharmaceutical Education and Research, Borgaon (Meghe), Wardha - 442 001, India

**Keywords:** Metoprolol tartrate, carbopol, bioadhesion, swelling study, *in vitro* release

## Abstract

Buccoadhesive tablet of metoprolol tartrate was developed to prolong its release and improve bioavailability by avoidance of hepatic first pass metabolism during the treatment of chronic hypertension. The formulations were tested for weight, hardness, friability, content uniformity, swelling index, bioadhesive force and drug release rate. Carbopol 934 P was used as bioadhesive polymer and methocel K4M was added as a matrix former. Backing layer of ethyl cellulose was given to the tablets. Optimised formulation containing carbopol 934 P and methocel K4M in the ratio of 1:1 showed surface pH values in the range of 6 to 7 and 91.50% cumulative release of drug in 10 h. Stability study revealed that the optimized formulation was stable for atleast 3 mo.

The potential of buccal mucosal route of drug administration was first recognized by Walton and others reported in detail on the kinetics of buccal mucosal absorption^1^–^3^. Buccal delivery of drugs provides an attractive alternative to the oral route of drug administration particularly in overcoming deficiencies associated with the later mode of dosing. Problems such as high first pass metabolism, and drug degradation in the harsh gastrointestinal environment, can be circumvented by administering the drug via the buccal route. Moreover buccal drug delivery offers a safer method of drug utilization, since drug absorption can be promptly terminated in cases of toxicity by removing the dosage form from the buccal cavity^4^.

Metoprolol tartrate is a selective β_1_ adrenergic antagonist^5^. It is reported to lack intrinsic sympathomimetic activity and to have little or no membrane stabilizing activity and is widely used to treat essential hypertension^6^. Metoprolol is readily and completely absorbed from the gastrointestinal tract but is subjected to considerable first pass metabolism and half life is 3 to 4 hours^7^. Hence it is a suitable candidate for administration via the buccal route. Nielsen and Rassing have shown that metoprolol tartrate has the permeability value of 165 × 10 ^−6^ cm/s across TR 146 cell culture model which can be used as an *in vitro* model for permeability studies of human buccal drug delivery^8^.

The objective of present study is to design buccoadhesive bilayered tablets which will release the drug unidirectionally in buccal cavity for extended period of time in order to avoid first pass metabolism for improvement in bioavailability, to reduce the dosing frequency and to improve patient compliance.

Metoprolol tartrate (Ipca Laboratories Ltd. Ratlam), Carbopol 934 P (Ruger Chemicals Co. Inc. Irvington, NJ) and Methocel K4M (Colorcon Asia Pacific Pvt. Ltd. Singapore) were used. All other chemicals, either reagent or analytical grade, were used as received.

Buccoadhesive tablets were fabricated by the direct compression method using the formulae shown in Table 1. All the ingredients were blended in mortar with a pestle to obtain uniform mixing. The blended powder of the core layer was lightly compressed on 8 mm flat faced punch (Cadmach, single punch tablet compression machine), the upper punch was removed and ingredient of backing layer was added over it and finally compressed at a pressure that gave a Monsanto hardness of 10 to12 kg/cm^2^. Each tablet weighed approximately 250 mg with a diameter of 8 mm.

**TABLE 1:** COMPOSITION OF METOPROLOL TARTRATE BUCCOADHESIVE TABLETS

Ingredients (mg)	Formula code		
	
	F1	F2	F3
Metoprolol tartrate	100	100	100
Carbopol 934 P	50	25	75
Methocel K4M	50	75	25
Ethyl cellulose	50	50	50
Magnesium stearate	1	1	1
Polymer ratio	1:1	1:2	2:1

Formula for the preparation of buccoadhesive tablets of metoprolol tartrate

Tablets were evaluated for weight variation, hardness, friability and drug content uniformity. The dimensional specifications were measured using Vernier calipers. Hardness was determined using Monsanto hardness tester and friability test was performed by using Roche friabilator. Weight variation test and test for content uniformity was conducted as per specifications of IP 1996^9^,^10^. The swelling rate of buccoadhesive tablets was evaluated using a 1% w/v agar gel plate^11^. For each formulation 3 tablets were weighed and average weight of each 3 tablets was calculated (W1). The tablets were placed with the core facing the gel surface in 7 Petri dishes (each containing 3 tablets) which were placed in an incubator at 37 ± 0.1°. Three tablets were removed at time intervals of 0.5. 1, 2, 3, 4, 5 and 6 hour, excess water on the surface was carefully absorbed using filter paper and swollen tablets were weighed. The average weight (W2) was determined and then swelling index was calculated using the formula

% Swelling index = ((W2-W1)/W1)×100.

The surface pH of the tablets was determined in order to investigate the possibility of any side effects, on the oral cavity. As acidic or alkaline pH is found to cause irritation to the buccal mucosa, hence attempt was made to keep the surface pH close to the neutral pH. Buccoadhesive tablets were left to swell for 2 h on the surface of an agar plate. The surface pH was measured by means of pH paper placed on the core surface of the swollen tablet^12^,^13^. Bioadhesive strength of the tablets was measured on a modified physical balance using the method described by Gupta *et al.*^14^,^15^. Porcine buccal mucosa was used as the model membrane and phosphate buffer pH 6.8 was used as the moistening fluid.

The dissolution rates of the buccal tablets were studied using the USP I rotating basket method at 37±0.5° and 50 rpm. Tablets containing 100 mg metoprolol tartrate were added to 900 ml of phosphate buffer pH 6.8. Samples were withdrawn at certain time intervals and replaced with fresh dissolution medium. The amount of metoprolol tartrate released was determined spectrophotometrically at 275 nm. The release rate study was carried out for 10 h.

Further to characterize the release mechanism of metoprolol tartrate from buccoadhesive tablets, the dissolution data were evaluated according to the relationship proposed by Korsmeyer *et al.*, as in following equation^7^,^11^, Mt/M_∞_ = kt^n^, where, Mt/M_∞_ is the fractional release of the drug, t denotes the release time, k- constant incorporating structural and geometrical characteristics of the device and *n*- diffusional exponent that characterized the type of release mechanism during the dissolution process. For non-Fickian release, the *n* values falls between 0.5 and 1, while in case of Fickian diffusion, *n* = 0.5; for case II transport, *n* =1, and for supercase II transport, *n* is greater than 1. Different kinetic equations were applied to interpret the release rate of metoprolol tartrate from the buccoadhesive tablets. The values of *n* as estimated by linear regression of log (Mt/M_∞_) versus log (t) and the coefficient of determination (R^2^) of all the three formulations are shown in Table 2.

**TABLE 2:** KINETIC ASSESSMENT OF RELEASE DATA (R^2^) AND ESTIMATED VALUES OF n AND k BY REGRESSION OF LOG (Mt/M_∞_) ON LOG (t)

Batch	Zero order kinetics	Square root t kinetics	n	k	r^2^
F1	0.9942	0.9965	0.5635	0.4950	0.9975
F2	0.9732	0.9962	0.6361	0.4213	0.9954
F3	0.9918	0.9967	0.6736	0.4322	0.9986

Coefficient of determination (R^2^) of formulations F1, F2 and F3

Stability study was carried out on the formulation F1. Tablets of batch F1 were wrapped in an aluminum foil then placed in an amber colored bottle. It was stored at 40°, 75±5% relative humidity for 3 months. Tablets were evaluated for physical characteristics, bioadhesion properties and *in vitro* drug release after every one month. Stability studies data obtained was compared with data obtained for zero time at ambient temperature. Results were analyzed using Student's Unpaired ‘t’ test.

All the tablets with different proportion of polymer composition were within the weight range of 250 to 251 mg. The hardness of all the tablets was found to be in the range of 10 to 12 kg/cm^2^. The loss in total weight of the tablet due to friability was in the range of 0.1 to 0.2%. The drug content in different tablet formulations was highly uniform and in the range of 99 to 102%. The results of the swelling study indicated that the tablets did not show any appreciable change in their shape and form during the 6 h they were kept on agar plate. The surface pH of F1 and F2 was found to be in the range of 6 to 7 indicating that it will not cause any irritation in the oral cavity. Formulation F3 had shown surface pH in the range 3 to 4 that might be due to presence of higher amount of carbopol 934 P, a polyacrylic acid polymer.

*In vitro* release profile for formulations F1, F2 and F3 are shown in fig. 1. Out of all the three formulations, formulation F3 exhibited the maximum drug release, i.e. 94.56% but as the surface pH of this tablet was in acidic range, formulation F1 was selected as optimized formulation. Cumulative % of drug release from F1 was found to be 91.50%. No statistically significant difference was obtained between cumulative percent of drug release from F1 and F3 (*p* > 0.05) using Student's Unpaired ‘t’ test.

**Fig. 1:**
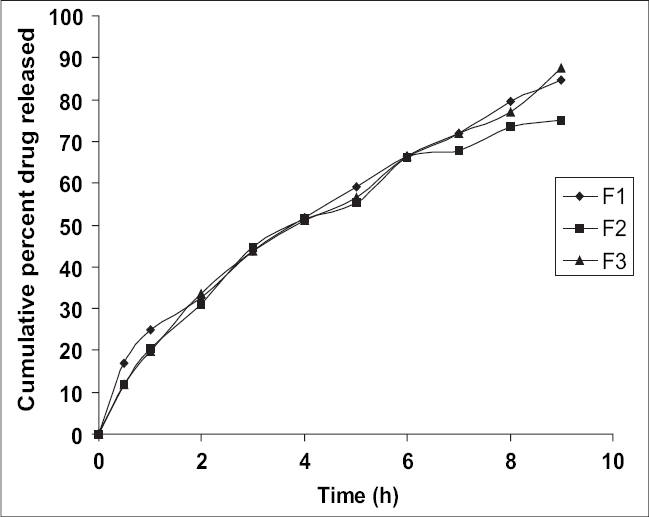
Plot of cumulative percent drug released *in vitro* versus time. Drug release from formulations F1 (♦), F2 (■) and F3(▲)

Carbopol 934 P is more hydrophilic than methocel K4M, it can swell rapidly, therefore decrease of carbopol 934 P content delays the drug release from formulation F2. The maximum cumulative percent release of metoprolol tartrate from formulation F3 could be attributed to the presence of higher amount of carbopol 934 P.

The obtained values of *n* (diffusion exponent) lie between 0.5 and 1.0 in all the formulations exhibiting a non-Fickian release behavior controlled by a combination of diffusion and chain relaxation mechanism. Results of kinetic data indicated that release rate from all the three formulations best fitted square root t kinetics.

The Bioadhesion characteristics were affected by the ratio of bioadhesive polymer (fig. 2). The highest adhesion force was possessed by formulation F3 (0.3499 N), i.e. formulation containing higher amount of Carbopol 934 P. Adhesion force of formulation F3 was followed by formulation F1 (0.2980 N) and formulation F2 (0.2632 N) which contains Carbopol 934 P and Methocel K4M in the ratio of 1:1 and 1:2, respectively. This indicated that the strength decreases as another polymer is mixed with polyacrylic acid^16^. Results of stability studies of formulation F1 indicated that it was stable at 40°, 75±5% relative humidity as there was no statistically significant difference observed for dissolution (*p* > 0.05) and bioadhesion data (*p* > 0.05).

**Fig. 2:**
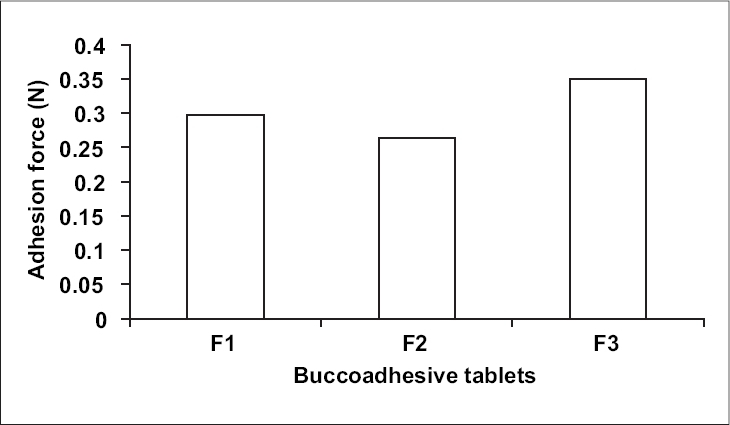
Bioadhesive strength of three formulations of metoprolol tartrate (*n* = 5)

From the entire study it can be conducted that the stable formulation could be developed by incorporating Carbopol 934 P and Methocel K4M in the ratio of 1:1 for sustaining the release of metoprolol tartrate from buccoadhesive tablets with adequate bioadhesiveness and swelling properties.
